# Hypoxia and ischemic stroke modify cerebrovascular tone by upregulating endothelial BK(Ca) channels—Lessons from rat, pig, mouse, and human

**DOI:** 10.1111/apha.70030

**Published:** 2025-03-21

**Authors:** Christian Staehr, Victoria Hinkley, Vladimir V. Matchkov, Rajkumar Rajanathan, Line Mathilde B. Hansen, Yvonne Eiby, Nathan Luque, Ian Wright, Stella T. Bjorkman, Stephanie M. Miller, Rohan S. Grimley, Andrew Dettrick, Kirat Chand, Hong L. Nguyen, Nicole M. Jones, Tim V. Murphy, Shaun L. Sandow

**Affiliations:** ^1^ Department of Biomedicine, Health Aarhus University Aarhus Denmark; ^2^ Department of Anaesthesiology and Intensive Care Aarhus University Hospital Aarhus Denmark; ^3^ Biomedical Science, School of Health University of the Sunshine Coast Maroochydore Queensland Australia; ^4^ UQ Centre for Clinical Research, School of Clinical Medicine, Faculty of Medicine University of Queensland St Lucia Queensland Australia; ^5^ Blacktown Clinical School, Faculty of Medicine Western Sydney University Blacktown New South Wales Australia; ^6^ Department of Physiology, School of Biomedical Sciences, Faculty of Medicine University of New South Wales Sydney New South Wales Australia; ^7^ School of Clinical Medicine, Faculty of Medicine James Cook University Townsville Queensland Australia; ^8^ Sunshine Coast University Hospital Birtinya Queensland Australia; ^9^ School of Medicine Griffith University Birtinya Queensland Australia; ^10^ Stroke and Ageing Research, Department of Medicine, School of Clinical Sciences at Monash Health Monash University Clayton Victoria Australia; ^11^ Department of Pharmacology, School of Biomedical Sciences, Faculty of Medicine University of New South Wales Sydney New South Wales Australia

**Keywords:** BK_Ca_, blood flow, endothelium, hypoxia, ion channel, stroke

## Abstract

**Aim:**

In animal models and human cerebral arteries, the changes in endothelial cell (EC)‐large conductance calcium‐activated potassium channel (BK_Ca_) distribution, expression, and function were determined in hypoxia and ischemic stroke. The hypothesis that hypoxia and ischemic stroke induce EC‐BK_Ca_ in cerebral arteries was examined.

**Methods:**

Immunohistochemistry analyzed BK_Ca_ expression in EC and smooth muscle (SM) of the middle‐cerebral artery (MCA) from rat, piglet, and mouse, and pial arteriole of human. Pressure myography with pharmacological intervention characterized EC‐BK_Ca_ and TRPV4 function in rat MCA. Electron microscopy determined caveolae density and vessel properties in rat and mouse MCA.

**Results:**

In rat, pig, and human cerebral vessels, EC‐BK_Ca_ was absent in normoxia; present after *chronic* (rat) and *acute* hypoxia (pig), post‐ischemic stroke in human vessels, and after endothelin‐1‐induced stroke in rats. Mouse MCA EC‐BK_Ca_ expression increased after *acute* hypoxia. In rat MCA post‐hypoxia and stroke, EC and SMC caveolae density increased, with reduced medial thickness, and unchanged diameter. Caveolae and BK_Ca_ did not colocalize. In rat MCA, iberiotoxin (IbTx) potentiated pressure‐induced tone in hypoxia/stroke, but not in normoxia. In normoxia, overall MCA tone was unaffected by endothelial removal, but was increased in hypoxia/stroke, where there was no additive effect of endothelial removal and IbTx on tone. Functional TRPV4 was expressed in EC of rat MCA post‐stroke.

**Conclusions:**

In post‐hypoxia/stroke, but not in normoxia, EC‐BK_Ca_ contribute to the regulation of MCA tone. Identifying unique up‐ and downstream signaling mechanisms associated with EC‐BK_Ca_ is a potential therapeutic target to control blood flow post‐hypoxia/stroke.

## 1 INTRODUCTION

Arterial endothelial cells (EC) regulate vascular tone and blood flow, with small and intermediate conductance calcium‐activated potassium channel (S/IK_Ca_) expression and function being key to their dilator and blood flow activity in health and disease.^1^, ^2^, ^3^ In a similar, but distinct manner, intact artery EC‐large (B)K_Ca_ expression and function can range from absent to a prominent role, depending on the artery, species, and state; including in disease.^4^ In vascular ECs, the BK_Ca_α pore‐forming and β1‐regulatory/auxiliary subunits are often (but not always) reported at a transcriptional mRNA level, and depending on the vascular bed, are not always translated to protein.^4^ This differential expression suggests a potential for rapid EC‐BK_Ca_ upregulation in specific beds and states, noting that EC isolation and culture induce BK_Ca_ expression.^4^

While EC‐BK_Ca_ are reported in a limited number of intact vascular beds, species, strains, and states, including in disease (Table 1), they are generally absent in control (e.g., normoxia) but can be present/upregulated by hypoxia (48 h, as *chronic* ^5^, ^6^, ^7^, ^8^), as it is shown in skeletal muscle arteries of adult Sprague–Dawley (SD) rats.^5^, ^6^, ^7^, ^8^ This upregulation is associated with a hyperpolarized smooth muscle (SM) membrane potential, impaired myogenic tone, and reduced contraction,^5^, ^6^, ^7^, ^8^ and thus increased dilation. In hypoxia, elevated EC‐BK_Ca_ may be an adaptive response to disease‐related vascular dysfunction. Increased vascular EC‐BK_Ca_ may thus be pertinent in states of reduced tissue oxygenation such as the occlusion of ischemic stroke, myocardial infarction, and pulmonary embolism. In the cerebral circulation, reduced oxygen has significant effects on brain function and anatomy, including core and penumbra development, typical of ischemic stroke.^9^

**TABLE 1:** Large conductance calcium‐activated potassium channels (BK_Ca_) occur in *intact* vessel endothelium of limited species, beds, and states.

Species/strain/sex (m/f)/age	Vessel type/form	Method/s	Refs.
Human			
n.s.	Internal mammary artery	IHC, myography, WB	[37]
n.s.	Internal thoracic artery	IHC, myography, q‐PCR, WB	[38]
m, 4; f, 4; 67 years	Mesenteric artery, 3rd order, colon cancer	rt‐PCR	[18]
m, 2, f, 5; 57 years	Absent in non‐cancer patients		
m, 22, f, 7; 66 years	Pulmonary artery	Myography	[39]
n.s.	Saphenous vein	IHC, q‐PCR, myography, WB	[38]
Mouse			
C57BL6 and *KO, male, 8–12 weeks	Coronary arteriole	IHC	[40]
Pig			
n.s.	Renal (conduit) artery	Myography	[41]
Rat			
Wistar, male, n.s.	Left anterior descending coronary artery	Myography	[42]
SHR, male, 7 mth	Mesenteric artery, superior, *and*	Myography, WB	[43]
WKY, male, 7 mth	Absent *in WKY*, superior	Myography, WB	[43]
Wistar, n.s.	Mesenteric artery, 1st order	Myography	[44]
SD, male, n.s.	Mesenteric artery, 3rd order	Myography	[45]
SD, n.s.	Mesenteric artery, 4‐5th order	Myography	[46]

Abbreviations: *KO, knock out, TRP ankyrin and vanilloid type 1; IHC, immunohistochemistry; m/f, male/female; mRNA, via rt‐/qPCR; n.s., not stated; SD, Sprague–Dawley; SHR, spontaneously hypertensive rat; WB, Western blot; WKY, Wistar Kyoto.

The distribution and function of specific components of spatially localized EC‐signaling microdomains, including aspects of S/I/BK_Ca_ and related calcium store and accessory proteins, can differ between species, beds, and states, including in disease. Such domains occur at non‐caveolae and/or caveolae‐associated membrane; the latter primarily being omega‐shaped invaginations ~80 nm at their widest point.^10^, ^11^ In ECs of intact gracilis muscle artery of chronic hypoxic SD rats, the transient receptor potential vanilloid type 4 (TRPV4) calcium channel can form a functional microdomain with BK_Ca_ and caveolin‐1, the caveolae‐linked eNOS scaffolding protein.^12^ Caveolae can be integral in regulating receptor, channel, and effector function and distribution,^10^, ^11^ including those associated with normal and altered vessel activity in hypoxia and stroke. In artery ECs and SMCs, caveolae may in part facilitate the differential compartmentalization of S/I/BK_Ca_ and TRPV4 channels, with this spatial localization facilitating key aspects of vascular dilator and constrictor signaling mechanism/s.^2^ Multiple factors can modulate BK_Ca_ expression and activity, and these include access to calcium, sensitivity to messengers including IP_3_ and GTP‐binding proteins, and reactive oxygen species,^13^ as well as oxygen tension, shear stress, growth factors, and hormones such as insulin.^14^

The significance of EC‐BK_Ca_ in the middle cerebral artery (MCA) of mouse, pig, and rat models, and human pial arterioles, was determined with a focus on hypoxia and ischemic stroke. The hypothesis that hypoxia‐ischemic stroke induces EC‐BK_Ca_ expression in intact MCA and pial arterioles, and that caveolae compartmentalize BK_Ca_, was examined. To test these proposals, a rat model of *chronic* hypoxia and ischemic stroke was assessed for MCA function, and for BK_Ca_ and TRPV4 expression. Cerebrovascular BK_Ca_ expression was also examined in acute hypoxia in piglet and mouse models, and to provide translational insight, in ischemic stroke patients. The data have implications for understanding how hypoxia affects cerebrovascular BK_Ca_ activity in EC and SMC function and blood flow.

## 2 RESULTS

### 2.1 *Chronic* (5 days) hypoxia and ischemic stroke induce BK_Ca_ expression and upregulate TRPV4 in the endothelium of *rat* distal MCA

**FIGURE 1:**
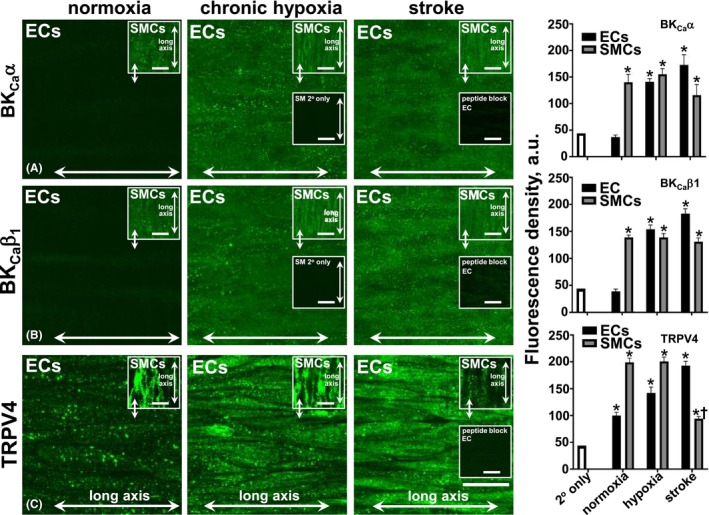
Immunohistochemical (IHC) distribution and expression of large conductance calcium‐activated potassium (BK_Ca_α and β1 subunits) and transient receptor potential vanilloid type 4 channel (TRPV4) in a distal middle‐cerebral artery from rats under normoxia, *chronic* (5 days) hypoxia and ischemic stroke conditions. Endothelial cell (EC)‐BK_Ca_α/β1 expression is absent in normoxia and upregulated in a diffuse and punctate manner in the cell membrane in chronic hypoxia and ischemic stroke (A, B, respectively). Diffuse and punctate BK_Ca_α/β1 occur on the smooth muscle cell (SMC) membrane of normoxia, hypoxia, and ischemic stroke (A, B, respectively, upper insets; green). Diffuse, intermittent punctate TRPV4 occurs in the ECs and SMCs of normoxia, hypoxia, and ischemic stroke (C; green), with density increased in hypoxia and stroke (C). Cell orientation relative to the longitudinal vessel axis indicates EC/SMC layer patency (A–C, and insets), with EC long axis, left to right. IHC insets (lower central panels in A, B, and far‐right semi‐quantitative panels in A–C) show staining with secondary antibody only, and with 10‐fold peptide excess blocking primary label (A–C, right IHC panels, lower insets). Double arrows, main and inset panels indicate the same vessel region, but different focal planes. Averaged fluorescence density values for tissue stained with secondary antibody only and with corresponding primary antibodies are shown in the bar graphs in the right panel. *n* = 6–8 each for normoxia, and 6 each for hypoxia and stroke, each from different animals. *p* < 0.05, cf. secondary only*, and normoxia^†^ cf. hypoxia or stroke. Bar, 25 μm.

Semi‐quantitative assessment of fluorescence density demonstrates differential BK_Ca_α and β1 appearance in ECs and SMCs of rat MCA (Figure 1). In ECs of normoxic MCA, both BK_Ca_α and β1 were absent, and their expression was upregulated after hypoxia and ischemic stroke (Figure 1A,B; *p* < 0.05). Conversely, no change in BK_Ca_α‐ and β_1_ expression was observed in the SMCs after hypoxia or ischemic stroke, compared to normoxic MCA (Figure 1A,B).

Semi‐quantitative fluorescence density shows increased EC‐TRPV4 expression after hypoxia and ischemic stroke, compared to normoxia (Figure 1C). SMC‐TRPV4 did not differ between normoxic and hypoxic conditions; although a decrease in stroke compared to matched control occurred (Figure 1C).

### 2.2 Ischemic stroke causes differential changes in caveolae density in endothelial cells of distal *rat* MCA

Conventional TEM determined gross characteristics of MCAs. The diameters of distal MCA were comparable between control and ischemic stroke rats (Table S3). However, after ischemic stroke, distal MCA SMC layers were reduced, associated with a thinner arterial wall compared to control.

The density and colocalization of the prevalent caveolae scaffolding coat protein (caveolin‐1) and BK_Ca_ in ECs and SMCs of the distal rat MCA were determined using TEM and immunoEM (Figure S1; Table S1). The density of lumenal and ablumenal caveolae in ECs and SMCs was increased in the distal MCA after ischemic stroke compared to control (*p* < 0.05; Table S3). ImmunoEM revealed BK_Ca_α expression in ECs of the distal MCA after ischemic stroke, in contrast to normoxic conditions where no endothelial BK_Ca_α was seen (Figure S1).

### 2.3 Ischemic stroke induces BK_Ca_ ‐dependent changes in the myogenic tone of the *rat* distal MCA

**FIGURE 2:**
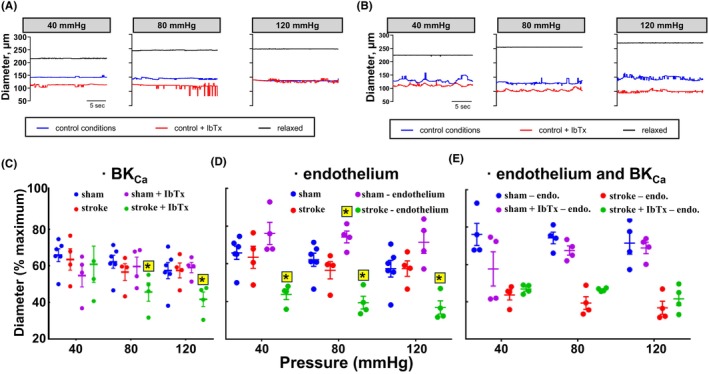
Pressurized distal middle cerebral artery (MCA) from control‐normoxia and hypoxia‐stroke rats developed myogenic tone. Representative traces for vascular diameter assessments of MCAs from sham‐operated rats (A) and after ischemic stroke (B). The same MCA diameter was assessed under control conditions, in the presence of IbTx, and as fully relaxed in calcium‐free solution at 40 mmHg, 80 mmHg, and 120 mmHg, as indicated. The vascular tone (C and D) was calculated as a percent of the active diameter of the fully relaxed diameter of the same artery. There was no difference in the amount of tone between sham and stroke MCA. However, while iberiotoxin (IbTx; BK_Ca_ inhibitor; 0.1 μM) had no effect on tone in the sham group (control, *n* = 6; IbTx, *n* = 4), it contracted MCA after stroke at 80 and 120 mmHg (C; *n* = 4, each for control and IbTx). Endothelial removal (air bolus) reduced tone in the sham group at 80 mmHg (*n* = 6, control; *n* = 4, endothelium removed) and increased tone at all pressures in stroke rats (*n* = 4; D). After endothelial removal, IbTx continued to have no effect on the tone of MCA from the sham group, but now had no effect on the MCA from stroke rats too (*n* = 4, for all) and on the endothelium‐denuded stroke group, in the absence and presence of IbTx (E). *significance of intervention (IbTx or ‐endothelium) on myogenic tone cf. control conditions, as *p* < 0.05, via two‐way ANOVA followed by Tukey's multiple comparisons test.

The myogenic tone of rat distal MCAs with intact endothelium at different transmural pressures demonstrated no difference between rats undergoing sham surgery and those with ischemic stroke at any given transmural pressure, for example, at 40, 80, and 120 mmHg (Figure 2A–C). This tone was stable and reproducible during pressure steps over at least 1 h of experimental duration (not shown) suggesting no time‐dependent changes in tone during pharmacological interventions. Incubation (10–20 min) with the BK_Ca_ inhibitor, iberiotoxin (IbTx; 0.1 μM) at 80 mmHg slightly constricted sham distal MCA (from 65.6 ± 1.8% to 58.9 ± 2.2% of maximal diameter, *p* = 0.0516, *n* = 6) and this effect was potentiated for arteries after ischemic stroke (from 62.1 ± 7.1% to 45.4 ± 4.7% of maximal diameter, *p* = 0.0019, *n* = 4). Thus, although no difference in MCA tone between stroke and sham was seen prior to IbTx incubation, in the presence of IbTx, the tone of distal MCAs from ischemic stroke rats was higher than in the sham group (*p* = 0.0227, 2‐way ANOVA; Figure 2A–C).

Air bubble‐induced EC removal reduced the tone of distal MCA from rats in the sham group at 80, but not 40 or 120 mmHg, compared to vessels from the same group with intact endothelium (Figure 2D). Conversely, EC denudation of distal MCAs from ischemic stroke rats increased tone at all three transmural pressures. Studies using bradykinin and 5‐hydroxytryptamine confirmed endothelial ablation and subsequent functional integrity of SMCs, respectively (Figure S2).

Following ischemic stroke, distal MCAs with intact endothelium also exhibited greater tone in the presence of IbTx at 120 mmHg compared to the baseline conditions (Figure 2E). This IbTx effect was not observed in distal MCAs in the sham group. Notably, the presence of IbTx did not change vascular tone at different transmural pressures in endothelium‐denuded distal MCAs of both sham and ischemic stroke rats (Figure 2E), as there was no additive effect.

### 2.4 Functional interaction of TRPV4 and BK_Ca_ in *rat* distal MCA

**FIGURE 3:**
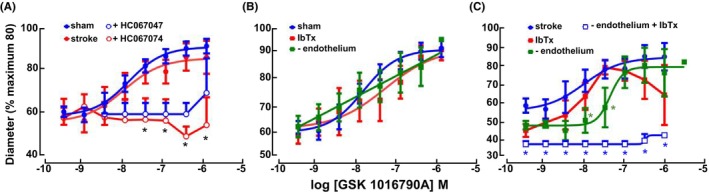
Distal middle‐cerebral artery (MCA) endothelial TRPV4 activity is increased after ischemic stroke. Effect of the TRPV4 activator GSK1016790A on the diameter of rat‐isolated, pressurized MCA from sham and stroke rats (*n* = 9, each) in the absence and presence of the TRPV4 antagonist HC067047 (0.3 μM; A; *n* = 4, each). Effects on MCA from sham (B; *n* = 6) and stroke rats (C; *n* = 6) following removal of the endothelium (B and C, *n* = 6 and 5, respectively) or in the presence of the BK_Ca_ blocker iberiotoxin (IbTx, 0.1 μM; B,C, *n* = 6, each), or removal of the endothelium and IbTx combined (C; *n* = 4). Data expressed as % of maximum diameter at 80 mmHg. *p < 0.05, two‐way ANOVA with Tukey's test. For (A), * indicates *p* < 0.05, as significance of HC067047 effect on response to GSK1016790A cf. MCA from sham and stroke rats, respectively, under control conditions. For (C), * indicates *p* < 0.05, as significance of the effect of endothelium removal (−endothelium) or of a combination of IbTx and endothelium denudation (−endothelium + IbTx) cf. responses of MCA from stroke rats under control conditions. Data compared with two‐way ANOVA followed by Tukey's multiple comparisons test. For pEC50 and Emax data, see Table S4.

The functional contribution of TRPV4 in rat distal MCA and its interaction with BK_Ca_ were assessed (Figure 3; Table S4). Relaxation to the TRPV4 agonist GSK1016790A (0.1‐1 mM) exhibited no difference between distal MCAs from sham and ischemic stroke rats (Figure 3A; Table S4). Pre‐incubation with the TRPV4 antagonist, HC‐067047, did not affect the vascular tone of MCAs from sham and ischemic stroke rats (from 65.7 ± 1.9% to 64.9 ± 3.5% and from 62.5 ± 2.8% to 57.7 ± 8.6%, respectively, *n* = 4) but abolished the GSK1016790A‐induced vasodilation in both groups (Figure 3A). The GSK1016790A‐induced MCA dilation in the sham group was unaffected by IbTx or endothelium denudation (Figure 3B; Table S4). However, after stroke, the GSK1016790A‐induced vasodilation was reduced after endothelium denudation (Figure 3C; Table S4). Moreover, vasodilation was nearly abolished by IbTx in the endothelial‐denuded arteries of ischemic stroke rats, suggesting a functional interplay between BK_Ca_ and TRPV4 channels in this state. However, the effect of functional antagonisms, that is, the suppression of GSK1016790A‐induced vasodilation because of increased myogenic tone, cannot be excluded.

### 2.5 *Acute* (30–50 min) hypoxia induces BK_Ca_α expression in the endothelium of *piglet* distal‐central MCA

**FIGURE 4:**
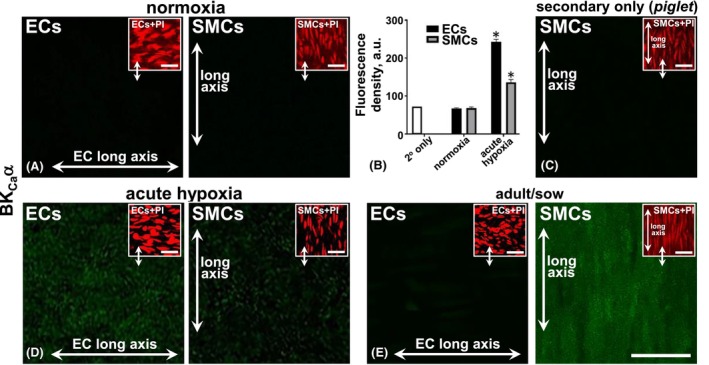
Immunohistochemical distribution and expression of BK_Ca_α in piglet central‐distal middle‐cerebral artery (c/dMCA) in normoxia and *acute* (30–50 min) hypoxia conditions. Whole‐mount MCA endothelium (EC) and smooth muscle cells (SMCs) do not express BK_Ca_ in normoxia (A) compared to the fluorescence density of the secondary antibody only (C). Diffuse‐punctate EC‐ and SMC‐BK_Ca_ expression occurred under hypoxia (D). Propidium iodide (PI; red) nuclear and cell orientation relative to the longitudinal vessel axis indicate EC/SM layer patency (A, C, D, E, insets), with the EC long axis, left to right. Antibody‐tissue controls as comparative adult MCA ECs and SMCs from normoxic adult/sow show negative EC and SMC positive signals (E). Double arrows in the main and inset panels (A, C, D, E) indicate the same vessel region and focal plane, but different labels. A 10‐fold peptide excess blocked the primary label (data not shown). Averaged fluorescence intensity data (B) for experiments as in representative images (A, C, D, E). *n* = 6 each, for normoxia and hypoxia piglet, and *n* = 3 for adult/sow, each from a different animal. *p < 0.05, as significant cf. normoxia and hypoxia, and hypoxia and secondary alone. Bar, 25 μm.

Semi‐quantitative fluorescence data show the absence of BK_Ca_α in ECs and SMCs of piglet MCAs under normoxic conditions (Figure 4). Following acute (30–50 min) hypoxia, BK_Ca_α expression was present in ECs and SMCs of the piglet central‐distal MCA compared to the normoxic control (Figure 4; *p* < 0.05; for vessel segment location, see Figure S3). Notably, for co‐incubated tissue, the SMCs of the adult sow MCA showed BK_Ca_α expression (Figure 4) as a positive control for the relatively low SMC expression in piglet MCA.

### 2.6 *Acute* (120 min) hypoxia induces BK_Ca_α expression in the endothelium of *mouse* distal MCA

**FIGURE 5:**
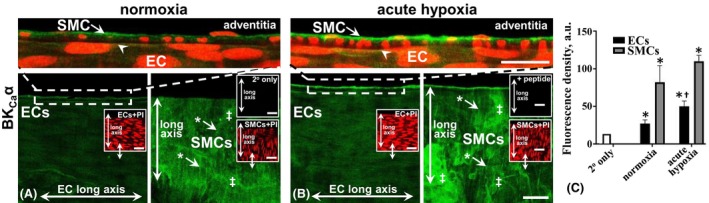
Immunohistochemical distribution and expression of BK_Ca_α in mouse distal middle‐cerebral artery in normoxia‐control and *acute* (120 min) hypoxia conditions. Whole‐mount vessel endothelium (EC) and smooth muscle cells (SMCs) express diffuse BK_Ca_α under normoxia (A) and hypoxia (B) conditions (green). Propidium iodide (PI; red) nuclear and cell orientation relative to the longitudinal vessel axis indicates EC/SM layer patency (A, B, insets), with EC long axis, left to right. Single (0.25 μm) ‘slices’ show brighter fluorescence at the SMC ablumenal surface in hypoxia cf. normoxia (dashed line boxes, cf. A,B); and also at the ablumenal over the lumenal SMC surface (A, B, cf. upper arrows and lower arrowheads). SMC ablumenal and lumenal surface fluorescence intensity is ~2.7 and 1.6‐fold increased in hypoxia cf. normoxia, respectively (*n* = 4; see also Figure S4; Table S5). Boxed regions of SM‐EC border/vessel edge (A, B, lower left panels) are enlarged (A, B, upper panels; see also Figure S3). In SMCs, a higher level localization of BK_Ca_α to cell borders occurs (A, B, lower right panels, e.g., arrows with asterisk), noting that these images are from the SMC‐adventitial border region; and hence, some pial‐related cell labelling^‡^). Staining with secondary antibody only (A, lower right panel, upper inset; C) and with 10‐fold peptide excess blocking primary label (B, lower right panel, upper inset). Double arrows, main and inset panels (A,B) indicate the same vessel region and focal plane, but different label. Averaged fluorescence intensity (C) as in representative images. *n* = 7 and 4, for normoxia and hypoxia, each from a different animal. *p* < 0.05, as significant cf. secondary only*, and normoxia^†^ cf. hypoxia EC, respectively. Bar, 25 μm.

Semi‐quantitative fluorescence data show BK_Ca_α in ECs and SMCs of mouse distal MCA under normoxic conditions and after *acute* (120 min) hypoxia (Figure 5, Figure S4). While BK_Ca_α was at a relatively low level in normoxia, acute hypoxia resulted in an increase in EC‐BK_Ca_α expression, while no significant change occurred in the SMCs. Notably, SMC‐BK_Ca_ expression was higher than that in ECs under both normoxic and hypoxic conditions (Figure 5, Figure S4).

When examining the edge of the distal MCA at higher magnification in the confocal images (Figure 5, Figure S4), SMCs‐BK_Ca_α expression was higher at the ablumenal (adventitial) compared to the lumenal cell surface. Overall, BK_Ca_α expression at both the ablumenal and lumenal SMCs was higher after acute hypoxia (Figure S4; Table S5).

Considering the distinctive differential focal spatial localization of BK_Ca_α at lumenal and ablumenal SMCs in the mouse distal MCA (Figure 5, Figure S4), caveolae density was determined at these sites in mouse MCA after normoxic and hypoxic conditions (Figure S3D,E, Table S5). In distal mouse MCAs, no difference in caveolae density was found between lumenal and ablumenal SMC membranes under normoxic and acute hypoxic conditions, or when comparing normoxic and hypoxic distal MCAs (Table S5).

### 2.7 Ischemic stroke induces BK_Ca_α expression in endothelium of *human* pial arteriole

Expression of BK_Ca_α was also assessed in cerebral arterioles of postmortem ischemic stroke patients and their matched controls. EC‐BK_Ca_α was absent in control, present in stroke, and in SMCs of control and stroke groups (Figure S5). Notably, given the inherent observation of absence and presence of EC‐BK_Ca_ in the human pial artery of control and hypoxia‐stroke (where both had time between death and collection), respectively, this is consistent with the interval having no role in the observed altered expression (of absence to presence, respectively).

## 3 DISCUSSION

The effect of acute and chronic hypoxia and ischemic stroke on BK_Ca_ expression in the ECs of MCA from mouse, pig, and rat, as well as human pial arterioles, was determined. Low level EC‐BK_Ca_ expression occurs in acute and is significantly higher in chronic hypoxia and ischemic stroke, whilst being absent (rat, pig, human) to low (mouse) in the normoxic group. The disparity in MCA EC expression across species in both normoxia and hypoxia may be due to differences in basic signaling pathways, including in the stimuli and local functional MCA environment. Indeed, the mechanism/s for de novo EC‐BK_Ca_ expression and function may also arise from differences in the model disease state, such as the length of the acute (30–50 and 120 min for pig and mouse, respectively) and chronic (5 days, rat) ischemia protocols, and the idiosyncrasies therein.

In rat post‐stroke MCA, the appearance of EC‐BK_Ca_ contributes to the regulation of myogenic tone, while in a similar manner, in in vivo mouse MCA, dilation increases with hypoxia, 24 h after ischemic stroke.^15^ Another study showed that increased endothelial‐derived hyperpolarization and nitric oxide pathways underlie amplified vasodilation of isolated rat MCA following asphyxical cardiac arrest.^16^ Thus, under hypoxia‐related pathological conditions, including ischemic stroke and cardiac arrest, potentiation of cerebrovascular dilation via increased EC‐BK_Ca_ expression is consistent with the mechanisms facilitating improved blood flow, at least in specific cerebral arteries and brain regions.

While BK_Ca_ mRNA and protein are predominantly absent in intact artery endothelium,^4^, ^17^ robust data show that select *mouse, pig*, and *rat* models and human arteries have EC‐BK_Ca_‐mediated function that can reflect BK_Ca_ expression^18^ (see also Table 1). In the same species, the present data show that EC‐BK_Ca_ can be upregulated in the cerebral vasculature by hypoxia. These data are consistent with the absence of rat skeletal muscle artery EC‐BK_Ca_ in normoxia and its appearance following hypoxia in gracilis and femoral vessels.^5^, ^6^, ^7^, ^8^ However, the specific mechanism/s underlying the upregulation of EC‐BK_Ca_ are unknown. Indeed, hypoxia may affect several vascular mediators such as reactive oxygen species and protein kinase C‐dependent modulation of BK_Ca_ function,^17^ altered growth factors,^14^ acid–base disturbances,^19^ the (transcription) hypoxia‐inducible factor (HIF) and related oxygen tension changes,^20^ as well as changed mechanosensory activity associated with altered blood flow.^21^ While there is significant overlap between these options, it is unknown whether SMC‐BK_Ca_ expression and function may or may not reflect that in EC‐BK_Ca_. If this is the case (at least in some states), as for example, in pulmonary artery SMCs, a low oxygen tension environment and related changes in HIF may enhance BK_Ca_‐mediated expression and dilation.^20^

Although the overall expression and distribution of SMC‐BK_Ca_ in MCA differ between rat, piglet, and mouse under normoxic conditions, SMC‐BK_Ca_α and β_1_ are unchanged by chronic hypoxia and ischemic stroke in rat or, for BK_Ca_α, by acute hypoxia in mouse. In mouse MCA, ablumenal SMC‐BK_Ca_α expression is higher than lumenal in both normoxia and hypoxia, as is expression in hypoxia over normoxia when determined separately at the lumenal and ablumenal sites. Similar to rat MCA EC‐BK_Ca_, this differential SMC‐BK_Ca_ expression and distribution imply a potential for related distinct SMC‐BK_Ca_ functional contribution; in this case at the ablumenal versus lumenal sites; although what activity this may confer is unknown. In contrast to the rat and mouse MCA, overall SMC‐BK_Ca_α expression is increased in the piglet after acute hypoxia; an issue that may relate to the immature developmental state of the piglets compared to the adult rat and mouse.^22^ Accordingly, in the MCA of adult (sow) pig, SMC‐BK_Ca_α was expressed, and is absent in EC.

While BK_Ca_α protein is absent in the endothelium of porcine and rabbit aorta, its corresponding mRNA is present.^23^, ^24^ At face value, this suggests an ability to facilitate rapid initiation of BK_Ca_ protein translation in ECs exposed to specific stimuli, such as occurs in disease, including hypoxia. In the present study, data from chronic compared to acute hypoxia in rats versus piglets and mice, respectively, suggest a potential quantitative association between the level of hypoxia and elevated MCA EC‐BK_Ca_α expression (compare EC‐MCA BK_Ca_ expression in Figures 1, 4 and 5 for rat, pig(let) and mouse, respectively).

In the present study, the expression and function of both TRPV4 and BK_Ca_ were upregulated in rat MCA after ischemic stroke, although EC‐TRPV4 did not directly activate EC‐BK_Ca_. However, the combination of endothelium denudation and BK_Ca_ inhibition had a strong synergistic effect in inhibiting TRPV4‐induced MCA dilation after stroke. This synergistic effect has to be further validated; potential functional antagonism of an elevated vascular tone for TRPV4‐induced vasodilation cannot be completely excluded. Thus, the TRPV4‐BK_Ca_ functional axis may contribute to the increased dilatory action of EC‐BK_Ca_ via a secondary mechanism associated with TRPV4 mediating EC‐Ca^2+^ influx and/or by generating the Ca^2+^ sparks that activate BK_Ca_, as occurs in SMCs.^25^ Interestingly, similarly to rat MCA SMC‐BK_Ca_ and SMC‐TRPV4 was unchanged by hypoxia, but in contrast, SMC‐TRPV4 was reduced after stroke compared to normoxia, suggesting a disparity in the regulation of EC and SMC‐TRPV4. The basal role of TRPV4 in mediating MCA EC Ca^2+^ is consistent with that of TRPC3 in facilitating EC‐Ca^2+^ influx in rat mesenteric artery^26^; although their activation of endogenous ligands, such as epoxyeicosatrienoic acids and α_1_‐adrenergic receptors via noradrenaline,^27^ remains to be determined.

The importance of caveolae, caveolins, and cavins (the key caveolae scaffolding and structural proteins), as membrane microdomain component responses to hypoxia, altered blood flow, and pressure is well recognized.^10^, ^28^, ^29^ Key factors include caveolae distribution, density, and composition; and transcriptional control of the key components. Several studies suggest that caveolae are a site, and in some cases imply the only site, for S/I/BK_Ca_ location in the ECs and/or SMC membrane in the arterial wall.^30^, ^31^ However, while there may be some association of caveolae and BK_Ca_, this is not universal, and immunoEM data in the present study suggest that BK_Ca_ are predominantly present at non‐caveolae sites in both EC and SMCs of MCA, as also inferred from the lack of correlation between caveolae and BK_Ca_ density, expression, and distribution. Hence, the present data suggest that EC caveolae density increases after chronic, but not acute, hypoxia, while BK_Ca_ expression increases under both conditions. Notably, channels such as BK_Ca_ and TRPV4 have been suggested to be confined to external regions of caveolae‐membrane, interacting with caveolin only when caveolae disassemble (Parton RG, pers. comm). Consistent with this, in *cultured* commercial passage 2–5 bovine aortic EC, the BK_Ca_ membrane current activation occurs exclusively in non‐caveolar membrane fractions.^32^ Of note, BK_Ca_ and caveolin‐1 have been suggested to colocalize in the SMCs of cremaster and the ECs of chronic hypoxic gracilis skeletal muscle arteries.^5^, ^31^ However, the confocal and immunoEM resolution used was insufficient to be conclusive on whether such ion channels and other signaling proteins occur *within* or *around* caveolae, or both, with further high‐resolution work required to clarify this.

## 4 MATERIALS AND METHODS

Detailed methods are available as Supporting Information.

Notably, to ensure that the use of a single experimental model did not bias the experimental findings and facilitate observation of potential similarities and differences in the signaling pathways therein to broaden data relevance, the study was performed in multiple species and experimental models.

## 5 LIMITATIONS

Targeting EC‐BK_Ca_ in MCA is a potential mechanism to facilitate cerebral blood flow control in specific disease states. However, as SMC‐BK_Ca_ expression and function are ubiquitous in adult arteries,^17^, ^25^ differentiating EC‐ and SMC‐BK_Ca_ targets when both are expressed is problematic, and thus control of EC‐BK_Ca_ in hypoxia/stroke is likely limited to modulating signaling pathways up‐ or downstream of its primary BK_Ca_ site of activity. Thus, subsequent studies will clarify the presence of distinct EC versus SMC signaling mechanisms as treatment targets, which include specific and distinct EC and SMC calcium modulation pathways, such as TRP and/or IP_3_R subtype activity, where EC‐, SMC‐, and vascular‐bed‐specific expression can occur.^33^, ^34^

An additional limitation relates to the ideal need to further examine the MCA in the acute hypoxia mouse and pig models in the chronic state to further clarify the progression of EC‐BK_Ca_ expression and function, per the present rat data. Indeed, the investigation of hypoxic effects on BK_Ca_‐EC expression and function in pulmonary and cardiac vessels, like that of the MCA, is also an area of interest and limitation, whereby hypoxia‐related injury may have critical pathophysiological significance for embolism and infarction, respectively.

A technical limitation of the present data relates to submembranous caveolae numbers, in that these may be overestimated due to the potential for membrane folding making enclosed caveolae‐like dimensions (appearing as ~80 nm diameter spheres^35^); thus, suggesting caution in interpreting that data set. Additionally, it would be ideal to record EC membrane potential in intact arteries, particularly in relation to determining the detailed aspects of artery EC dilator signaling in hypoxia and normoxia. However, unfortunately, the technical requirements for this are beyond the scope of the present study.

## 6 CONCLUSIONS

In the present study, both hypoxia and ischemic stroke induce EC‐BK_Ca_ expression in the MCA, contributing to the regulation of cerebrovascular myogenic tone to facilitate blood flow as an adaptive mechanism in disease. Modulation of EC‐controlled perfusion is an important focus for the development of treatments to improve outcomes after ischemic stroke and other cerebral hypoxia‐related disorders.^36^

## 7 THERAPEUTIC IMPLICATIONS

Modulating EC‐BK_Ca_ is a potential therapeutic target to control blood flow in hypoxic and ischemic stroke states; albeit, doing so directly is limited due to ubiquitous SMC‐BK_Ca_ expression. Hence, further focus on determining EC and SMC‐specific up‐ and downstream pathways related to BK_Ca_ control is a priority to develop therapy to control dilation and thereby mediate blood flow.

## AUTHOR CONTRIBUTIONS

Christian Staehr: (*equal); conceptualization; formal analysis; funding acquisition; investigation; methodology; project administration; resources; supervision; validation; visualization; writing—original draft preparation; writing—review and editing. Victoria Hinkley: (*equal); investigation; methodology; project administration; validation; visualization; writing—original draft preparation; writing—review and editing. Vladimir V. Matchkov: conceptualization; funding acquisition; investigation; methodology; project administration; resources; supervision; validation; writing—original draft preparation; writing—review and editing. Rajkumar Rajanathan: conceptualization; methodology; resources; writing—original draft preparation; writing—review and editing. Line Mathilde B. Hansen: investigation; methodology; resources; writing—original draft preparation; writing—review and editing. Yvonne Eiby: conceptualization; funding acquisition; methodology; resources; supervision; writing—review and editing. Nathan Luque: investigation; methodology; visualization; writing—original draft preparation; Ian Wright: methodology; resources; supervision; writing—review and editing. Stella T. Bjorkman: funding acquisition; methodology; resources; writing—review and editing. Stephanie M. Miller: funding acquisition; methodology; resources; writing—review and editing. Rohan S. Grimley: funding acquisition; writing—review and editing. Andrew Dettrick: funding acquisition; writing—review and editing. Kirat Chand: investigation; methodology; writing—original draft preparation; writing—review and editing. Hong L. Nguyen: conceptualization; investigation; methodology; project administration; writing—original draft preparation; writing—review and editing. Nicole M. Jones: conceptualization; investigation; methodology; project administration; resources; supervision; writing—original draft preparation; writing—review and editing. Tim V. Murphy: (lead; ^#^equal); conceptualization (lead; ^#^equal); formal analysis; funding acquisition; investigation; methodology; project administration; resources; supervision; validation; visualization; writing—original draft preparation; writing—review and editing. Shaun L. Sandow: (lead; ^#^equal); conceptualization; formal analysis; funding acquisition; investigation; methodology; project administration; resources; supervision; validation; visualization; writing—original draft preparation; writing—review and editing.

## FUNDING INFORMATION

Work was supported by the Brain Foundation (Australia), the University of the Sunshine Coast SPARK fund to S.L.S.; Lundbeck Foundation R344‐2020‐952, R412‐2022‐449, and Independent Research Fund Denmark 8020‐00084B to V.V.M.; and Riisfort, Knud Højgaard's, Helga & Peter Korning's (472123‐006 #55), Augustinus (#21‐2471), and Dagmar Marshall Foundations, Danish Cardiovascular Academy (PDC5‐2024004‐DCA; funded by the Novo Nordisk Foundation, grant number NNF20SA0067242, and the Danish Heart Foundation), and Gerhard Brønsted's travel grant to C.S.

## CONFLICT OF INTEREST STATEMENT

None.

## PATIENT CONSENT STATEMENT

Human experiments were approved by institutional review boards and participants gave informed consent.

## Supporting information


Data S1.

## Data Availability

The data that support the findings of this study are available from the corresponding author upon reasonable request.
