# Supplementary material for: Hypoxia and ischemic stroke modify cerebrovascular tone by upregulating endothelial BK(Ca) channels—Lessons from rat, pig, mouse, and human
Source: Acta Physiol (Oxf). 2025 Mar 21;241(4):e70030. doi: 10.1111/apha.70030 (PMC11926774; doi:10.1111/apha.70030)
Supplement: Supplementary file 1 — Data S1. [file APHA-241-e70030-s001.docx]

**Supporting Information**

**Detailed Methods**

**Ethics**

The care, handling and treatment of animals were in accordance with the Australian code of practice for the care and use of animals for scientific purposes (8^th^ edition, 2013) and the guidelines from directive 2010/63/EU of the European parliament on the protection of animals used for scientific purposes. The mice experiments were approved by the animal experiments inspectorate of the Danish ministry of environment and food, and reported in accordance with ARRIVE guidelines (Animal Research: Reporting of *in vivo* Experiments). Murine tissues were collected under approval from the Animal Experiments Inspectorate of the Danish Ministry of Environment and Food, 2019-15-0201-00341; porcine tissues were under approvals of the Universities of Queensland UQCCR/426/14/NHMRC, 2019/AE000534 and UQCCR/215/18, and Sunshine Coast ANA22196; and rat and human tissues under approval of the University of New South Wales ACEC 16/74B and HREAP HC210762/HREC HC200026, respectively.

**Tissue collection and experimental interventions**

A chronic rat hypoxia/ischemic stroke, and acute mouse and pig hypoxia models characterize endothelial cell (EC) BK_Ca_ expression and function in distal middle cerebral artery (MCA). Pial arteriole samples from ischemic stroke and non-stroke human pial arteriole samples also had their EC-BK_Ca_ profile clarified.

To ensure that use of a single experimental model did not bias the experimental findings, studies were performed in multiple species and experimental model systems. The use of these diverse species, the combination of data derived from male (rat) and equal numbers of male and female piglets and mice, using chronic (rat) and acute (piglet and mouse) models of hypoxia and stroke facilitates observation of potential similarities and differences in the signalling pathways therein, thus broadening relevance of the data. This approach alludes to key common underlying aspects of the signalling pathways involved, including facilitating identification of cell-type specific mechanisms for control of tone after hypoxia-stroke, to clarify both distinct and common signalling pathways influencing hypoxia-induced EC-BK_Ca_ expression in each of the acute and chronic models.

*Rat.* Male [Sprague Dawley](https://www.criver.com/products-services/find-model/cd-sd-igs-rat) (SD) rats, 8-10 weeks old were obtained from the Animal Resources Centre (Perth, Australia). The endothelin (ET)-1 (Auspep, Australia)-induced ischemia model was used, as previously.^1^ In brief, under isofluorane (1.5%) in oxygen anaesthesia by inhalation, a guide cannula (23 gauge) was implanted 1 mm dorsal to the right MCA. Rectal temperature was maintained at 35-37°C throughout surgery. Seven days following cannula implantation, conscious rats were subject to saline infusion (group **i**.) or MCA occlusion by ET-1 (120 pM in 6 μl of saline over 6 min) administration via a 30-gauge injector (group **ii**.). Twenty-four hours after saline infusion, a subset of rats was exposed to *chronic hypoxia* (**iii.**) by placing animals in a sealed plastic 9000 cm^3^ chamber (Biospherix; RRID:SCR_021177) with daily exposure (1 h /d for 5 d) to an 8% O_2_ and 92% N_2_ mixture via a Pro-Ox oxygen controller (Biospherix; RRID:SCR_021112)^1^. Gas flow was maintained at 200 ml/min and no more than four rats were placed in the chamber at any time.

Animals were monitored, scored and used based on their response to MCA-ET-1 occlusion, as stroke behaviour and severity.^1,2^ In brief, altered behaviour as an index of severity was scored from 1-5, with circling clockwise or counter-clockwise, forelimb clenching or dragging on the contralateral side to the occluded artery, assessed. Animals without behavioural indicators were excluded, while saline infused animals were used as sham treatment (per above).

A total of three groups were examined for immunohistochemistry (IHC); **i.** saline infused normoxia; **ii.** ET-1 infused stroke, and **iii.** saline-infused hypoxia. Two groups were used for transmission electron microscopy (TEM) and myography (**i.** normoxia and **ii.** stroke), and one as stroke (**ii**) for immunoEM.

Five days after experimental interventions, rats were deep anaesthetized (150 mg/kg pentobarbitone, i.p.). For IHC, rats were perfusion-fixed with 4% paraformaldehyde in PBS; or otherwise perfused with saline. Brains were removed and the ipsilateral MCAs distal to the occlusion site (**Figure S3A,B**) isolated for subsequent work.

*Pig.* An equal number of male and female White-Landrace piglets (*Sus scrofa domesticus*; University of Queensland piggery, Gatton) ≤24 h old, weighing 1.6 ± 0.2 kg, were equally assigned to normoxia and *acute hypoxia* groups. Additional control tissues from 3 adult sows, ~225 kg each, were examined.

Piglets were intubated and ventilated, and sedated using a loading dose of propofol (0.5 mL/kg) through a cannulated ear vein (24 gauge, BD Insyte cannula) and anaesthesia maintained with a continuous infusion of 10 mg/kg/h (propofol, 9 mg/ml)/alfentanil, 50 µg/mL). Fluids (10% glucose at 3 ml/kg/h) were administered via a cannulated mammary vein (24 gauge).

Hypoxia was induced by reducing the fraction of inspired oxygen (FiO_2_) to 4% and adjusting as necessary (2–6%) to achieve and maintain low-amplitude electroencephalography (EEG <5 µV) for 30 min.^3^ The hypoxic insult was sustained for 30 min or until arterial blood gas parameters (arterial pH <7.0, or arterial base excess [ABE] <–12 mM) were achieved. Hypotension (mean arterial blood pressure [MABP] <30 mmHg) was maintained for a period >10 min. The insult was titrated to individual animal physiological responses to achieve moderate-to-severe survivable brain injury,^3^ requiring a maximum ~50 min insult. Sham piglets underwent all procedures and anaesthesia without exposure to hypoxia.

Anaesthesia was ceased at the end of the insult period, and animals gradually transitioned off ventilation, and subsequently extubated 1-2 h post-hypoxic insult, and euthanised with an intraperitoneal injection of sodium pentobarbitone (325 mg/kg) at 72 h post-insult. Piglets were perfused intracardially with phosphate-buffered saline (PBS) and brains quickly dissected, coronally sliced into 3 mm sections and hemisected. Segments with the central (ipsilateral) MCA were isolated and immersion fixed in 4% paraformaldehyde, PBS overnight at 4**°**C. The MCA segments (~20 mm) were carefully dissected out and further fixed in 4% paraformaldehyde in PBS.

*Mouse.* An equal number of male and female C57BL6 mice, aged 8-12 weeks were used (Janvier, Le Genest-Saint-Isle, France). Mice were anesthetized with 2% isoflurane in 21% O_2_ and 79% N_2_. An initial midline cervical incision was applied to guide the intubation, as previous.^4^ Ventilation frequency was adjusted (model 845, Harvard Apparatus, Holliston, MA, USA) to maintain a stable end-tidal CO_2_ of 3.5% measured by a capnograph (Type 340, Harvard Apparatus). The body temperature was kept at 37°C (50-7222F, Harvard Apparatus).

Gas composition was controlled using a gas mixer (GMS instrument, Viborg, Denmark). The normoxic group was kept on 21% O_2_ for 120 min. The *acute hypoxia* group was gradually reduced from 21% to 7% over 90 min and kept at 7% for the last 30 min, with its composition confirmed (ABL 80, Radiometer, Denmark). Mice were subsequently heparinized, and perfusion fixed with 4% paraformaldehyde in PBS, and segments with the (ipsilateral) MCA isolated.

*Human* pial arterioles were collected from 3 each of control and ischemic stroke subjects from Sydney Brain Bank stocks that met the criteria of; 1. MCA with single or multiple infarct regions. 2. An intact pial surface. 3. Not fully cavitated, and 4. Clinically neurologically asymptomatic (i.e. no evidence of neurodegenerative disease on records/histology), apart from the stroke deficit (**Table S1**), with tissue taken from the primary ischemic / occluded (ipsilateral) side. Unaffected control tissue from the inferior parietal gyrus were from as closely as available age and sex-matched individuals (per **Table S1**). Formalin fixed (4%), paraffin-embedded tissue of known stereotaxic coordinates were cut as 5 μm thick sections.

**Pressure myography and pharmacological intervention in rat distal middle-cerebral artery (MCA)**

Rats were deeply anaesthetized with 100 mg/kg pentobarbitone (i.p.), brains removed and ~0.5 mm long segments of distal MCA, downstream of the ET-1/saline injection site and free of branches (**Figure S1A-C**), dissected, cleaned of surrounding tissue and placed in modified Krebs solution (in mM: 111 NaCl, 25.7 NaHCO_3_, 4.9 KCl, 2.5 CaCl_2_, 1.2 MgSO_4_, 1.2 KH_2_PO_4_, 11.5 glucose and 10 HEPES) in the chamber of a pressure myograph. Both ends of the MCA segments were secured to glass cannulas with nylon suture. The segments were pressurized, without intralumenal flow, and tested for leaks by ensuring the ability to maintain pressure at 120 mmHg for 30s while the pressure pump was inactive, and then stretched to optimal length at this pressure. Successfully mounted segments were then superfused with Krebs solution (3 ml/min, 37°C) and equilibrated at 40 mmHg before the pressure was raised to the working value of 80 mmHg. Arterial inner diameter was measured using edge-detection software (DiamTrak) or calibrated video callipers; in some vessels where vasomotion was observed, mean diameter over a period relevant to the vasomotion (20-30 seconds) was obtained using LabChart8.

The pressure-diameter relationships were established at 80, 40 and 120 mmHg. At each pressure the artery segment was held at the test-pressure for 10 min prior to measurement. Subsequently, pressure was returned to 80 mmHg and concentration-response relationship established to the TRPV4 activator GSK1016970A (1 nM-1 μM;^5-8^). Arteries were then incubated with either the BK_Ca_ inhibitor iberiotoxin (0.1 μM;^6,9-11^) or the TRPV4 inhibitor HC-067047 (0.3 µM;^5,7,8^) for 15 or 30 min, respectively; before re-evaluating the pressure-diameter and concentration-response relationships for the TRPV4 activator (at 80 mmHg). At the end of the experiment, arteries were superfused in the bath with a nominally Ca^2+^-free Krebs solution (lacking CaCl_2_ and with 2 mM EGTA) for 20 min to obtain the maximum passive diameter at 80 mmHg.

Functional denudation of the endothelium was performed at the very beginning of the relevant experiments by passing an air bolus through the pressurized segment. Successful denudation was confirmed by lack of dilatory response to bradykinin (30 and 100 nM;^12,13^). Vessel function post-denudation was also tested by examining the response to 5-hydroxytryptamine (1 µM).

**Immunohistochemistry**

The distribution of BK_Ca_ was examined in the central region of the MCA in normoxic (*n*=7) and hypoxic mice (*n*=4), piglets (*n*=6 each, for normoxic and hypoxic animals), and rat (*n*=6-8, 6 and 6, for normoxic, hypoxic and ischemic stroke, respectively) using conventional whole-mount confocal IHC, as previously.^14^

In brief, fixed whole-mount tissues were pinned on Sylgard in a 30-chamber multi-well plate, where diameter was >~150 μm, the MCA lumen was opened-up as a flat sheet (cutting one side of the artery ‘tube’, parallel to the long vessel axis), to facilitate EC imaging over the most-flat region available. Tissue was then incubated in blocking buffer (PBS containing 1% bovine serum albumin, 0.1% Triton-X detergent) for 2 h at room temperature (RT), rinsed (3 x 5 min) in PBS and incubated in primary antibodies (**Table S2**) in blocking buffer for 18 h at 4°C. The samples were then rinsed (3 x 5 min) in PBS and incubated in 1:100 in species specific secondary antibody, diluted in PBS containing 0.01% Triton X detergent for 2 h at RT. The samples were subsequently rinsed (3 x 5 min) in PBS; mounted in buffered anti-fade glycerol and cover slipped. To verify cell layer patency, propidium iodide (0.002%) in buffered glycerol was added to selected samples. Vessels were examined with a Nikon Eclipse Ti (RRID:SCR_021242) or Olympus FV3000 (RRID:SCR_017015) confocal microscopes and images acquired with uniform settings from initially blinded tissue preparations. Based on the assumption that relative fluorescence (RFD) and protein density have a semi-quantitative association, the confocal fluorescence signals were determined using the integrated density-measurement log function in Photoshop (RRID:SCR_014199). The semi-quantitative analysis involved selection of 4 equally sized randomly selected regions of interest from each preparation.

Notably, given that artery smooth muscle cell (SMC) BK_Ca_ is ubiquitous and relatively highly expressed in the vascular media,^15,16^ but was found to be relatively low in ~24 h old piglet MCA, a developmental change was suggested and hence, adult (2-3 yr old) sow MCA was used as a comparative positive control for SMC expression.

Human pial arteriole sections were dewaxed and hydrated, rinsed (3 x 5 min) in PBS and incubated in 2% hydrogen peroxide for 3 min, blocked with 10% ‘blotto’ non-fat milk (Santa Cruz) ~18 h at 4^o^C, and incubated in primary antibody (**Table S2**) for 4 h at ~22^o^C. Sections were subsequently rinsed (3 x 5 min) in PBS with 0.1% Triton-X and incubated in HRP conjugated secondary (**Table S2**) for 2 h at ~22^o^C, rinsed in PBS (3 x 5 min) and incubated in DAB-peroxidase. Sections were counterstained with hematoxylin and eosin and permount coverslipped.

Antibody controls involved the use of positive and negative expressing tissue, peptide block and Western blotting, as previously.^9,10,17,18^ Details are provided in **Table S2**, including example citations for antibody specificity.

**Conventional electron microscopy**

Tissue preparation for EM was as previous.^9,10^ In brief, MCA from mice and rats were dissected from anaesthetized animals, and fixed in 1% paraformaldehyde, 3% glutaraldehyde in 0.1 mM sodium cacodylate buffer, with 10 mM betaine, pH 7.4, embedded in Araldite 502. The MCAs were sectioned perpendicular to their long vessel axis and imaged in a JEOL 1100 transmission electron microscope at 10-60 k at 16 MP. Quantitative measurements of wall properties were made from vessel cross sections from ultrastructural montages taken at 1.5-2.5k at a 16 MP resolution. ImageJ/FIJI (RRID:SCR_003070) and Photoshop were used for measurements, with the stitch tool applied on edges of adjacent images in montages; only where caveolae were absent. Brightness and contrast enhancement were applied to EM images using Photoshop.

On the low magnification montages, diameter was determined by measuring the length of the circular internal elastic lamina. The number of SMC layers was determined from profiles ≥5 μm in length, averaging 4 regions 90^o^ apart for each *n*, and wall thickness at the same regions.^10^

Caveolae were defined as omega-shaped membrane invaginations, as ‘true’ caveolae, ~80 nm at their widest point, and ‘enclosed’ submembranous caveolae, of a similar-size, but as apparent vesicles, within 200 nm of the EC surface were also noted.^9,14^ Caveolae density was counted from 3-7 randomly selected 5-20 μm long regions of EC or SMC membrane, from four sites per MCA, and averaged as an individual ‘*n*’. Lumenal caveolae counts were from the EC or SMC side facing the lumen and vice versa for ablumenal.

**Immunoelectron microscopy**

To optimize ultrastructural and antigenic preservation, high pressure freezing, automated freeze substitution and low temperature embedding of fresh rat MCA in Krebs buffer was performed.^19^ In brief, unfixed fresh MCA segments from rats, heavily anaesthetized with 44/8 mg kg ketamine/xylazine, were frozen at high pressure (∼2100 bar; Leica EM HPF; RRID:SCR_021367), freeze-substituted at -90°C using a Leica freeze substitution device in 0.2% uranyl acetate in acetone for 4 d. Samples were then infiltrated and embedded in LR White (ProSciTech) at -25°C, and polymerized under UV light in the freeze substitution unit (RRID:SCR_020230) at -25^o^C. Sections were then mounted on formvar (0.5% in chloroform) and carbon-coated (~5-10 nm) slot grids, incubated in blocking buffer (as above, for IHC) for 30 min, followed by primary antibody (**Table S2**) in blocking buffer for 2 h at 22^o^C, followed by 10 nm Au-conjugated secondary antibody (**Table S2**) in 0.01% Tween 20 for 2 h at RT. Antibody specificity was confirmed per IHC, as above. Sections were imaged in a JEOL 1100 transmission electron microscope at 10-60 k at 16 MP.

**Reagents.**

Unless otherwise stated reagents were from Merck.

**Statistics**

Unless specified, results are expressed as means ± SEM. All/each ‘*n*’ are independent samples/biological replicates, in that each experiment was performed in one artery from one animal only. Active diameter is expressed as a percentage of the passive diameter observed in nominally Ca^2+^-free Krebs solution. The negative log of the half-maximal effective concentration (pEC50) was calculated by nonlinear regression analysis of individual concentration-response curves, noting related maximum dilation (Emax; GraphPad Prism v10; RRID:SCR_002798). Data sets satisfied the Shapiro-Wilks test for normality (α=0.05) and were subsequently analyzed using a two-way ANOVA with Tukeys multiple comparisons for individual groups of data or if warranted, a student *t*-test (GraphPad Prism). *P*<0.05 was considered significant. For IHC, multiple measures for individual preparations were averaged representing *n*=1 for each preparation, with each from a different animal / person, and data expressed relative to secondary antibody only.

For sample size calculations, we utilized the software G Power and compared the level of a continuous variable in two independent groups in a repeated measures *ANOVA* with a type I error rate of α = 0.05 (5%) and power of 1-β > 0.8 (80%) based on preliminary experiments and prior studies. For example, for ~30% change in expression, response or myogenic tone, we calculated a sample size of *n* = 4 for each group would be sufficient with an effect size of *d* = 1.30.

**Supplemental Figures and Figure Legends**

**
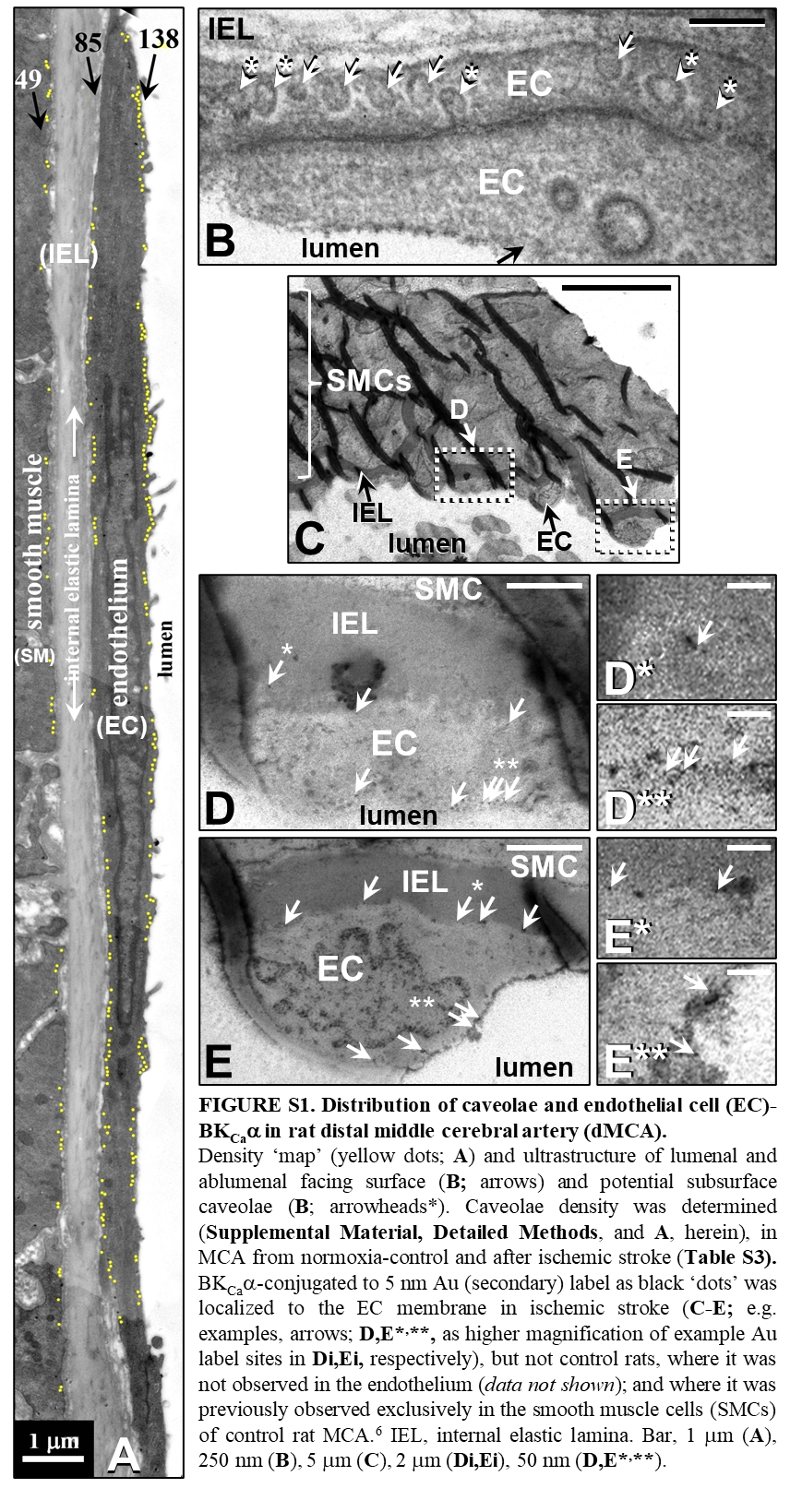
**

**Figure S2. 5-HT-induced constriction of isolated, pressurized (80 mmHg) middle cerebral artery, with and without endothelium, from sham-operated rats and rats after ischemic stroke.** *significant constriction, compared with appropriate sham operated group (*P*<0.05, ANOVA followed by Sidak’s test). Neither stroke nor removal of the endothelium impaired the ability of the vessels to respond to 5-HT.

**
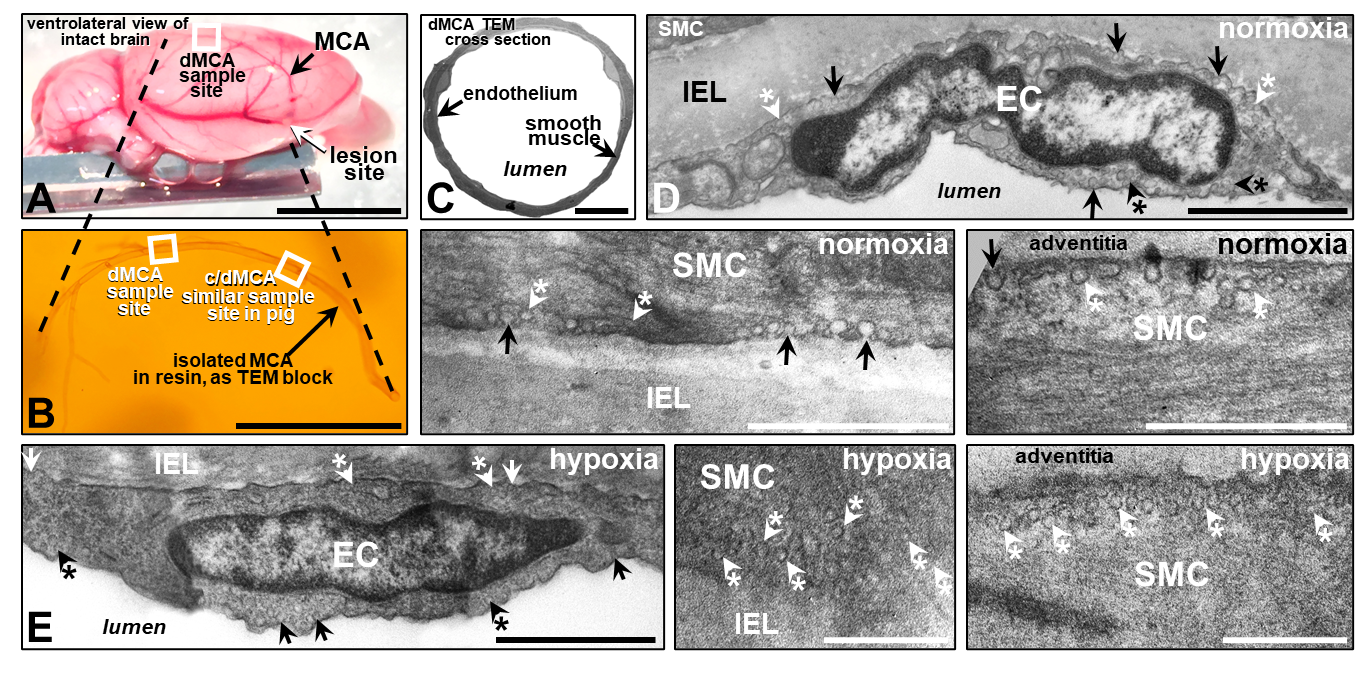
**

**FIGURE S3.** **General rat (A) and mouse (B,C) brain / middle-cerebral artery (MCA) morphology, and mouse distal (d)MCA endothelial caveolae distribution (D-I).**

Ventrolateral view of rat brain post-ischemic stroke/endothelin-1 treatment with dMCA sample (box) and lesion (white arrow) sites highlighted (**A**), with isolated (similar) mouse dMCA sample site and ultrastructural dMCA cross-section (region in left box, **B**; and pig sample site as right box, c/dMCA), with endothelial cells (ECs) lining the lumen, surrounded by smooth muscle cell (SMC) layers (**C;** ~160 μm diameter). Dashed lines linking **A-B** indicate similar vessel morphology in rat and mouse. Ultrastructure of lumenal and ablumenal facing surface (**D,E;** arrows) and potential subsurface caveolae (**D,E**; arrowhead*) from mouse dMCA. Caveolae density was determined per **Supplemental Material, Detailed Methods** in dMCA from normoxic (**D**) and hypoxic (**E**) mice (**Table S5**); *n*=4 and 3, respectively, each from different animals. IEL, internal elastic lamina. Bar, **A,B**, 10 mm; **C**, 50 μm; **D,E**, 1 μm.

**
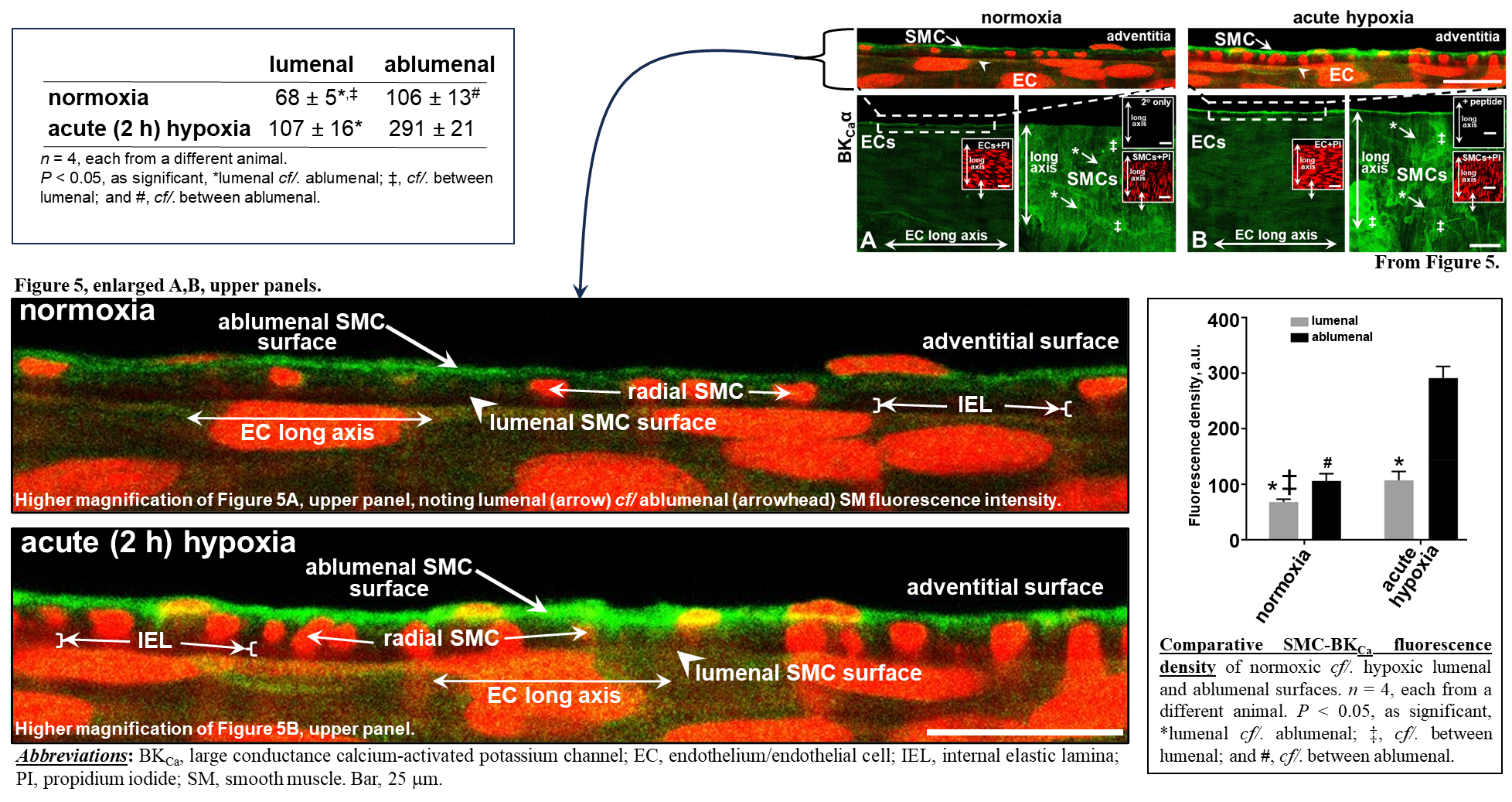
**

**FIGURE S4. Comparative SMC-BK_Ca_ fluorescence density at distal MCA, as higher magnification of Figure 5A,B, upper panels.**


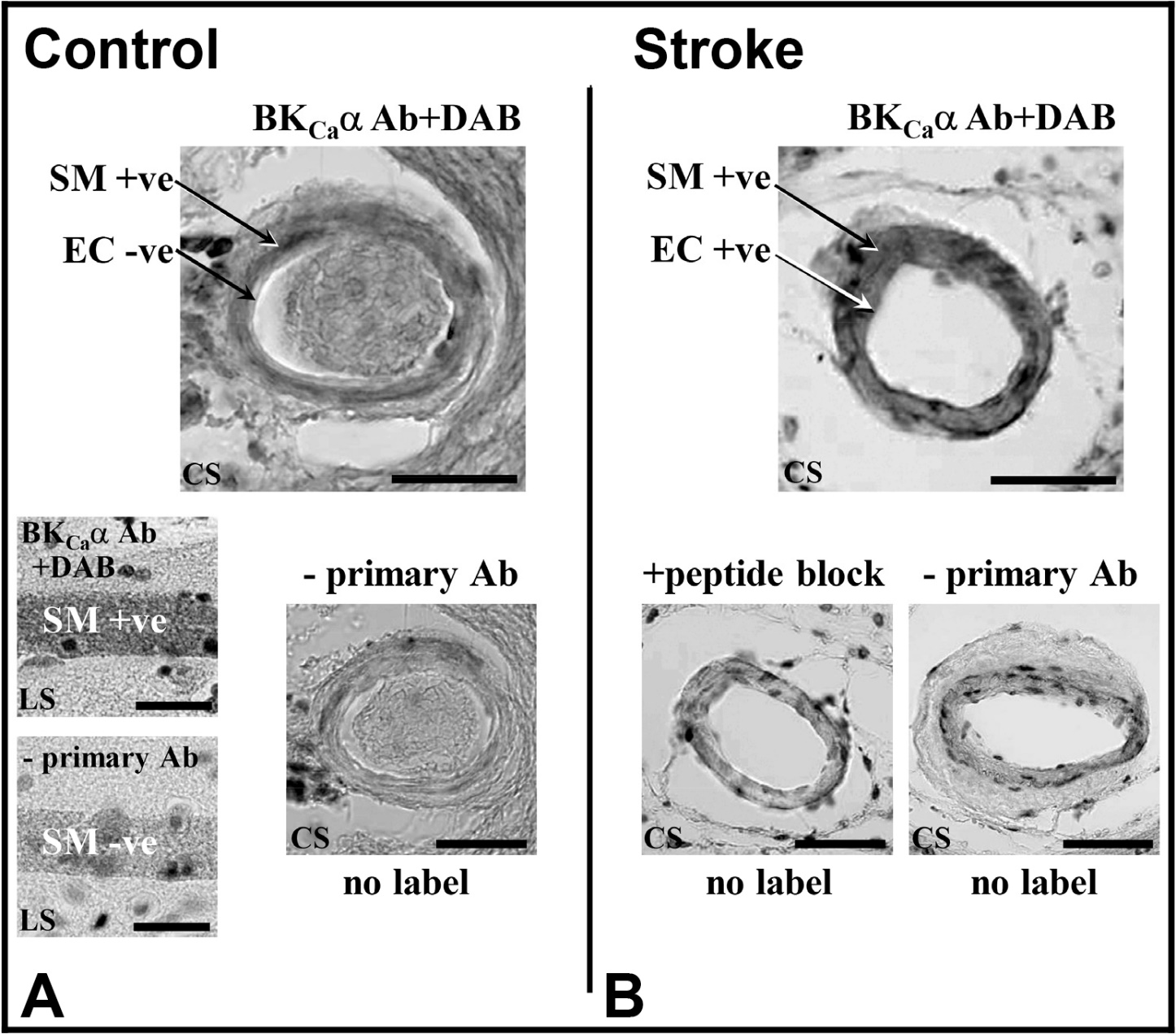


**FIGURE S5. Diaminobenzidine-peroxidase immunohistochemical distribution and expression of large conductance calcium-activated potassium channel (BK_Ca_α) in human pial arteriole from control and ischemic stroke subjects.**

Endothelial cell (EC)-BK_Ca_α was absent in control (**A**), present in stroke (**B**), and in smooth muscle (SM) of control and stroke (**A,B**, upper panels). Positive label is indicated by black diaminobenzidine (DAB)-peroxidase precipitant (**Supplemental Material, Detailed Methods**). Peptide block and absence of primary result in no labelling (**A,B**, lower panels). *n* = 3, each for control and ischemic stroke patients (characteristics, per **Table S1**). Ab, antibody; CS, cross section; LS, longitudinal section. Bar, 50 μm.

**Supplemental Tables**

**TABLE S1.** **Control and ischemic stroke patient characteristics of pial arteriole source.**

| **case**  **characterization** | **duration**  **of multiple**  **strokes (yrs)** | **cause of**  **death** | **age**  **(yrs)** | **gender** | **postmortem**  **delay; death to tissue collection (h)** |
| --- | --- | --- | --- | --- | --- |
| control | 0, not applicable | pneumonia | 100 | female | 24 |
| control | 0, not applicable | renal failure | 68 | male | 11 |
| control | 0, not applicable | sepsis | 89 | male | 24 |
| MCA infarct, inferior parietal gyrus | 20 | pneumonia | 88 | female | 6 |
| MCA infarct, inferior frontal gyrus | 25 | lung cancer | 69 | male | 32 |
| MCA infarct, insular cortex | 16 | cardiorespiratory failure | 88 | male | 23 |

MCA, middle cerebral artery.

**TABLE S2**. Antibody characteristics.

| antibody (Ab) | amino acid epitope / *research resource identifiers (*RRID) | species raised in | [supplied] / [working] | supplier / catalog, batch number/s | peptide available with Ab | homology | accession | specificity / characterization citation/s^ii.^ |
| --- | --- | --- | --- | --- | --- | --- | --- | --- |
| 1. BK_Ca_α / anti-KCNMA1 / K_Ca_1.1 | murine 1184-1200; RRID:AB_2040091 | rabbit | 0.6 mg/ml / 1:100 | Alomone APC-107; lots AN07, APC107, AN0825 | yes | 100% to rat; 16/17, bovine, chicken, dog, human | [Q08460](http://www.uniprot.org/uniprot/Q08460) | ^10,20^  - *knock-out verified.* |
| 2. peptide | (C)STANRPNRPKSRESRDK intracellular loop near C’ | synthetic | 0.4 mg/ml / 1:10 excess | Alomone BLP-PC107 | to above antibody | - as above. | [Q08460](http://www.uniprot.org/uniprot/Q08460) | - as above. |
| 3. BK_Ca_α / anti-KCNMA1 / K_Ca_1.1 | rat 199-213; RRID:AB_10915895 | rabbit | 0.8 mg/ml / 1:100 | Alomone APC-151; lot APC151AN0525 | yes | 14/15, human, mouse, rat | [Q62976](http://www.uniprot.org/uniprot/q62976) | ^21,22^ |
| 4. - peptide | (C)DSSNPIES(S)QNFYKD,  1^st^ extracellular loop | synthetic | 40 μg / 1:10 excess | Alomone BLP-PC151 | to above antibody. | - as above. | [Q62976](http://www.uniprot.org/uniprot/q62976) | - as above. |
| 5. BK_Ca_β1 / anti-sloβ1 / KCNMB1 | bovine 118-13 | rabbit | serum / 1:1000 | Merck, Garcia^i.^ | serum only | - | - | ^10^ |
| 6. BK_Ca_β1 / anti-sloβ1 / KCNMB1 | rat 2-17; RRID:AB_2040095 | rabbit | 0.8 mg/ml / 1:100 | Alomone APC-036, lot APC036AN0502 | yes | 100% to dog, human, mouse, rabbit | [P97678](http://www.uniprot.org/uniprot/p97678) | ^21,23^; *noting limitations raised by*^17,24^ |
| 7. peptide | KKLVMAQKRGETRALC, intracellular near N’ | synthetic | 0.4 mg /ml / 1:10 excess | Alomone BLP-PC036 | to above antibody. | - as above. | [P97678](http://www.uniprot.org/uniprot/p97678) | - as above. |
| 8. TRPV4 / OTRPC4 | rat 853-871, CDGHQQGYAPKWRAEDAPL; RRID:AB_532289 | rabbit | 0.8 mg/ml / 1:100 | Sigma T9075, batch / lot SLCN7200 | no | 17/19, mouse’ 16/19, human | [Q9ERZ8](http://www.uniprot.org/uniprot/Q9ERZ8) | ^7^ |
| 9. TRPV4 / OTRPC4 | human derived to a 20 amino acid; intracellular epitope; RRID:AB_592927 | rabbit | 1 mg/ml / 1:100 | Lifespan Biosciences  LS-A8583 | yes | 100% to bovine, human, dog, hamster, horse, pig rat. 19/20, rabbit, 18/20 chicken | [Q9HBA0](https://www.uniprot.org/uniprot/q9hba0) | ^18,25^ |
| 10. donkey anti-rabbit IgG CF™ 633 | - | rabbit | 2 mg/ml / 1:100 | Sigma-Merck SAB4600132, lots 20C1006 and 21C0928 | - | - | - | - |
| 11. HRP-goat anti-rabbit IgG | - | rabbit | 1:100 | Biorad, 9701107 | - | - | - | - |
| 12. 5 nm Au rabbit anti-goat IgG | RRID:AB_259951 | rabbit | 14.5 μg/ml / 1:40 | Sigma, G7277 | - | - | - | - |

i. From Merck Research Laboratories, NJ, U.S.A.

ii. Citation denotes inclusion of characterization data, comparative to tissue of known positive or negative expression; and not just previous use.

**Antibody issues / caveats.**

*In terms of the use of antibodies and the data in the present study, please note that;*

- The Merck and Alomone BK_Ca_β1 antibodies (**Table S2, rows 5 and 6**) showed similar, but not the same labelling (**Figure 1D-F**, Merck data only shown), with the latter showing increased signal, noting definitive reports of its non-selective / non-specific affinity for BK_Ca_β1 (see citations in e.g.^17,24^) albeit it is still available (Alomone, APC-036 / peptide, BLP-PC036); and thus produces potentially misleading results when used as the exclusive source for antibody-based detection / distribution and expression data of this regulatory BK_Ca_β1 protein.

- The Sigma and Lifespan TRPV4 antibodies (**Table S2, rows 8 and 9**) showed the same labelling properties, consistent with their previous use and characterization.

- Noting the use of characterized antibodies in this study and the above limitation in Alomone β1 specificity and use, additional verification of protein expression data would be of value. However, while IHC allows differentiation of EC and SM expression (noting BK_Ca_ is ubiquitously expressed in the latter^15,16^), other methods to achieve this are problematic, in that they require isolation of EC from SM; whereby (per **Introduction**) doing so induces EC BK_Ca_ expression.^17^

**TABLE S3.** **Rat distal middle-cerebral artery and caveolae properties in normoxia and ischemic stroke.**

| **cohort and ‘*n*’** | **normoxia (*4*)** | **stroke (*4*)** |
| --- | --- | --- |
| diameter (μm) | 136 ± 5 | 146 ± 9 |
| number smooth muscle (SM) layers | 2.8 ± 0.2 | 2.0 ± 0.2* (↓ ~30%) |
| wall thickness (μm) | 13.6 ± 0.1 | 7 ± 1.2* (↓ ~50%) |
| endothelial cell (EC) lumenal caveolae density / μm | 1.60 ± 0.12 | 2.93 ± 0.21* (↑ x~83%) |
| EC ablumenal caveolae density / μm | 1.12 ± 0.04 | 1.42 ± 0.05* (↑ x~26%) |
| SM lumenal caveolae density / μm | 0.74 ± 0.04 | 1.06 ± 0.02* (↑ x~43%) |

Mean ± SEM; **P*<0.05, significant compared to normoxic. ‘*n*’ (parenthesis), each for a different animal. For parameter clarification, see **Supplemental Methods**.

**TABLE S4. pEC_50_ and maximum dilation (Emax) values for GSK1016790A-induced dilation of pressurized MCA (80 mmHg) isolated from control and stroke rats.**

|  | **pEC50** | | | | |
| --- | --- | --- | --- | --- | --- |
|  | **Con** | **+ HC067047** | **+ IbTx** | **- endo** | **- endo + IbTx** |
| control | 7.91 ± 0.19 | NC | 7.38 ± 0.27 | 7.69 ± 0.30 | ND |
| stroke | 8.03 ± 0.43 | NC | 8.18 ± 0.26 | 7.38 ± 0.21* | NC |
|  | **Emax (% maximum diameter)** | | | | |
|  | **Con** | **+ HC067047** | **+ IbTx** | **- endo** | **- endo + IbTx** |
| control | 89.6 ± 3.4 | 69.0 ± 7.8* | 89.6 ± 3.6 | 93.3 ± 1.2 | ND |
| stroke | 84.6 ± 5.7 | 59.0 ± 6.3* | 74.4 ± 3.9 | 79.0 ± 5.8 | 42.6 ± 4.4* |

Mean ± SEM; **P*<0.05, significant, as drug or endothelium removal *cf/.* ‘Con’. *n*=5-9, each from a different animal, per **Figure 3**. endo, endothelium; IbTx, iberiotoxin; ND, not determined; NC, not calculable.

**TABLE S5.** **Mouse distal middle cerebral artery caveolae density (via TEM / μm) and BK_Ca_α relative fluorescence density (RFD to 2^o^ only) in normoxia and *acute* (120 min) hypoxia at lumenal and ablumenal SM membrane.**

| **cohort and ‘*n*’ (TEM)** | **normoxia (*4*)** | **hypoxia (*4*)** |
| --- | --- | --- |
| lumenal caveolae / μm | 7.69 ± 1.51 | 8.20 ± 0.78 |
| ablumenal caveolae / μm | 6.05 ± 0.45 | 4.75 ± 1.39 |
| **cohort and ‘*n*’ (immunohistochemistry)** | **normoxia (*4*)** | **hypoxia (*4*)** |
| lumenal BK_Ca_α RFD to 2^o^ only | 68 ± 5*^#^ | 107 ± 16^#^ |
| ablumenal BK_Ca_α RFD to 2^o^ only | 106 ± 13* | 291 ± 21 |

Mean ± SEM; *P*<0.05, significant, *normoxic *cf/.* to hypoxic; and ^#^lumenal *cf/.* to ablumenal. ‘*n*’ (parenthesis), each for a different animal.

**Supplemental References**

1. Nguyen HL, Ruhoff AM, Fath T, Jones NM. Hypoxic postconditioning enhances functional recovery following endothelin-1 induced middle cerebral artery occlusion in conscious rats. *Exp Neurol*. 2018;**306**:177-89.

2. Roulston CL, Callaway JK, Jarrott B, Woodman OL, Dusting GJ. Using behaviour to predict stroke severity in conscious rats: post-stroke treatment with 3', 4'-dihydroxyflavonol improves recovery. *Eur J Pharmacol*. 2008;**584**:100-10.

3. Bjorkman ST, Foster KA, O'Driscoll S M, Healy GN, Lingwood BE, Burke C, Colditz PB. Hypoxic/Ischemic models in newborn piglet: comparison of constant FiO_2_ versus variable FiO_2_ delivery. *Brain Res*. 2006;**1100**:110-7.

4. Staehr C, Giblin JT, Gutiérrez-Jiménez E, Guldbrandsen HO, Tang J, Sandow SL, Boas DA, Matchkov VV. Neurovascular uncoupling is linked to microcirculatory dysfunction in regions outside the ischemic core following stroke. *J Am Heart Assoc*. 2023;**12**:e029527.

5. Zhang P, Sun C, Li H, Tang C, Kan H, Yang Z, Mao A, Ma X. Transient receptor potential vanilloid 4 mediates endothelium-dependent contractions in the aortas of hypertensive mice. *Hypertension*. 2018;**71**:134-42.

6. Naik JS, Walker BR. Endothelial-dependent dilation following chronic hypoxia involves TRPV4-mediated activation of endothelial BK channels. *Pflugers Arch*. 2018;**470**:633–48.

7. Saxena A, Bachelor M, Park YH, Carreno FR, Nedungadi TP, Cunningham JT. Angiotensin II induces membrane trafficking of natively expressed transient receptor potential vanilloid type 4 channels in hypothalamic 4B cells. *Am J Physiol* 2014;**307**:R945-55.

8. White JP, Cibelli M, Urban L, Nilius B, McGeown JG, Nagy I. TRPV4: Molecular Conductor of a Diverse Orchestra. *Physiol Rev*. 2016;**96**:911-73.

9. Howitt L, Grayson TH, Morris MJ, Sandow SL, Murphy TV. Dietary obesity increases NO and inhibits BK_Ca_-mediated, endothelium-dependent dilation in rat cremaster muscle artery: association with caveolins and caveolae. *Am J Physiol*. 2012;**302**:H2426-76.

10. Howitt L, Sandow SL, Grayson TH, Ellis ZE, Morris MJ, Murphy TV. Differential effects of diet-induced obesity on BK_Ca_β1-subunit expression and function in rat skeletal muscle arterioles and small cerebral arteries. *Am J Physiol*. 2011;**301**:H29-40.

11. Riddle MA, Hughes JM, Walker BR. Role of caveolin-1 in endothelial BK_Ca_ channel regulation of vasoreactivity. *Am J Physiol* 2011;**301**:C1404-14.

12. Kohler R, Degenhardt C, Kuhn M, Runkel N, Paul M, Hoyer J. Expression and function of endothelial Ca^2+^-activated K^+^ channels in human mesenteric artery: A single-cell reverse transcriptase-polymerase chain reaction and electrophysiological study in situ. *Circ Res*. 2000;**87**:496-503.

13. Brakemeier S, Eichler I, Knorr A, Fassheber T, Kohler R, Hoyer J. Modulation of Ca2+-activated K+ channel in renal artery endothelium in situ by nitric oxide and reactive oxygen species. *Kidney Int*. 2003;**64**:199-207.

14. Grayson TH, Chadha PS, Bertrand PP, Chen H, Morris MJ, Senadheera S, Murphy TV, Sandow SL. Increased caveolae density and caveolin-1 expression accompany impaired NO-mediated vasorelaxation in diet-induced obesity. *Histochem Cell Biol*. 2013;**139**:309-21.

15. Hill-Eubanks DC, Werner ME, Heppner TJ, Nelson MT. Calcium signaling in smooth muscle. *Cold Spring Harb Perspect Biol*. 2011;**3**.

16. Krishnamoorthy-Natarajan G, Koide M. BK channels in the vascular system. *Intl Re Neurobiol*. 2016;**216**:401-38.

17. Sandow SL, Grayson TH. Limits of isolation and culture: intact vascular endothelium and BK_Ca_. *Am J Physiol*. 2009;**297**:H1-7.

18. Senadheera S, Bertrand PP, Grayson TH, Leader L, Murphy TV, Sandow SL. Pregnancy-induced remodelling and enhanced endothelium-derived hyperpolarization-type vasodilator activity in rat uterine radial artery: transient receptor potential vanilloid type 4 channels, caveolae and myoendothelial gap junctions. *J Anat*. 2013;**223**:677-86.

19. Senadheera S, Kim Y, Grayson TH, Toemoe S, Kochukov MY, Abramowitz J, Housley GD, Bertrand RL, Chadha PS, Bertrand PP, Murphy TV, Tare M, Birnbaumer L, Marrelli SP, Sandow SL. Transient receptor potential canonical type 3 channels facilitate endothelium-derived hyperpolarization-mediated resistance artery vasodilator activity. *Cardiovasc Res*. 2012;**95**:439-47.

20. Hei H, Gao J, Dong J, Tao J, Tian L, Pan W, Wang H, Zhang X. BK knockout by TALEN-mediated gene targeting in osteoblasts: KCNMA1 determines the proliferation and differentiation of osteoblasts. *Mol Cells*. 2016;**39**:530-5.

21. Balderas E, Torres NS, Rosa-Garrido M, Chaudhuri D, Toro L, Stefani E, Olcese R. MitoBK_Ca_ channel is functionally associated with its regulatory beta1 subunit in cardiac mitochondria. *J Physiol*. 2019;**597**:3817-32.

22. Lu M, Li JR, Alvarez-Lugo L, Li Y, Yu S, Li X, Shi B, Chai TC. Lipopolysaccharide stimulates BK channel activity in bladder umbrella cells. *Am J Physiol* 2018;**314**:C643-53.

23. Shi L, Liu X, Li N, Liu B, Liu Y. Aging decreases the contribution of MaxiK channel in regulating vascular tone in mesenteric artery by unparallel downregulation of α- and β1-subunit expression. *Mech Ageing Dev*. 2013;**134**:416-25.

24. Bhattarai Y, Fernandes R, Kadrofske MM, Lockwood LR, Galligan JJ, Xu H. Western blot analysis of BK channel β1-subunit expression should be interpreted cautiously when using commercially available antibodies. *Physiol Rep*. 2014;**2**: e12189.

25. Moayedi Y, Michlig S, Park M, Koch A, Lumpkin EA. Localization of TRP channels in healthy oral mucosa from human donors. *eNeuro*. 2022;**9**.
